# A Multidrug Donor Preconditioning Improves Steatotic Rat Liver Allograft Function and Recipient Survival After Transplantation

**DOI:** 10.3389/ti.2024.13557

**Published:** 2024-12-13

**Authors:** Min Xu, Salamah M. Alwahsh, Myung-Ho Kim, Otto Kollmar

**Affiliations:** ^1^ Department of General, Visceral, and Pediatric Surgery, University Medical Center Göttingen, Göttingen, Germany; ^2^ Liver Center, Massachusetts General Hospital, Harvard Medical School, Boston, MA, United States; ^3^ Department of Gastroenterology and Endocrinology, University Medical Center Göttingen, Göttingen, Germany; ^4^ Program of Medicine, College of Medicine and Health Sciences, Palestine Polytechnic University, Hebron, Palestine; ^5^ Department of Internal Korean Medicine, Woosuk University Medical Center, Jeonju, Republic of Korea; ^6^ Clarunis, Department of Visceral Surgery, University Centre for Gastrointestinal and Liver Diseases, University Hospital Basel, Basel, Switzerland

**Keywords:** allograft function, donor shortage, rat steatotic liver donor, ischemia reperfusion injury, multidrug donor preconditioning, orthotopic liver transplantation

## Abstract

The scarcity of donors has prompted the growing utilization of steatotic livers, which are susceptible to injuries following orthotopic liver transplantation (OLT). This study aims to assess the efficacy of multidrug donor preconditioning (MDDP) in alleviating injuries of steatotic grafts following rat OLT. Lean rats were subjected to a Western-style diet with high-fat (HF) and high-fructose (HFr) for 30 days to induce steatosis. Both lean and steatotic livers were implanted into lean recipients fed with a chow diet after OLT. The HF + HFr diet effectively elevated blood triglyceride and cholesterol levels and induced fat accumulation in rat livers. Our results demonstrated a significant decrease in alanine aminotransferase levels (*p* = 0.003), aspartate aminotransferase levels (*p* = 0.021), and hepatic Suzuki scores (*p* = 0.045) in the steatotic rat liver allograft group following MDDP treatment on post-operation day (POD) 7. Furthermore, the survival rates of steatotic rat liver allografts with MDDP (19/21, 90.5%) were significantly higher than those in the steatotic control (12/21, 57.1%, *p = 0.019). These findings indicate that MDDP treatment improves steatotic rat liver allograft function and recipient survival following OLT.

## Graphical Abstract

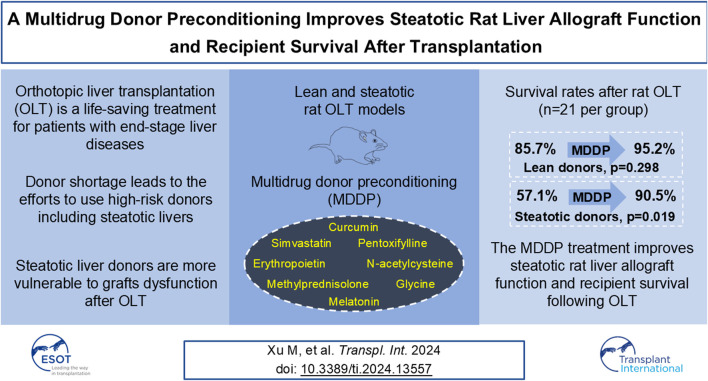

## Introduction

Orthotopic liver transplantation (OLT) stands as a crucial life-saving intervention for individuals suffering from end-stage liver disease. However, its widespread application is constrained by the scarcity of donors, a challenge exacerbated by the growing number of patients awaiting transplantation [1]. To alleviate this issue, marginal donors, including those with steatotic livers, advanced age, or prolonged ICU stays, have been increasingly considered for transplantation [2]. Nonetheless, these marginal donors are particularly susceptible to ischemia-reperfusion injury (IRI), which triggers the generation of free radicals upon liver reoxygenation, leading to lipid peroxidation and hepatocellular damage, with cold ischemia contributing to endothelial cell injury [3].

Metabolic dysfunction-associated steatotic liver disease (MASLD) has become a prevalent global health concern, propelled by factors such as sedentary lifestyles and consumption of high-fat and fructose-rich diets [4]. Western societies exhibit a heightened risk of MASLD due to dietary habits characterized by high fat and glucose intake, with fructose recently implicated in the development of necrotic inflammation and fibrosis in nonalcoholic steatohepatitis [5]. MASLD prevalence ranges from 10% to 30%, with rates soaring to 75%–92% among obese populations [6]. The escalating incidence of hepatic steatosis in the general populace is mirrored in the pool of potential liver donors. However, due to heightened vulnerability to hypoxia-reperfusion injury, hepatocytes laden with fat present a considerable challenge when utilized as donor allografts [7, 8]. Various animal models, such as the Lieber-DeCarli diet model [9] and fructose model [10–12], have been employed to study MASLD.

Multidrug treatment strategies have demonstrated efficacy in managing complex conditions such as HIV, cancer, and ischemic injury. These approaches have been extended to recondition liver donors to mitigate hepatic fat content and IRI ahead of transplantation. Over the past decades, several agents have been incorporated into perfusion and preservation solutions to reduce IRI risk in fatty liver donor rat OLT models, including melatonin [13], Treprostinil [14], carvedilol [15], cyclic RGD peptide [16], IL-6 [17], among others. Additionally, a multidrug cocktail comprising curcumin, simvastatin, N-acetylcysteine, erythropoietin, pentoxifylline, melatonin, glycine, and methylprednisolone has exhibited promise in diminishing IRI in fatty liver donors *in vitro* liver machine perfusion studies [18, 19]. Furthermore, the multidrug treatment approach has been implemented to decrease rat hepatic fat content during *ex vivo* normothermic machine perfusion for potential implantation [20, 21]. This indicates a promising method to broaden the liver donor pool by facilitating the utilization of steatotic livers while mitigating associated risks.

In this study, rats were fed either a standard chow diet or a Western-style diet rich in high-fat and fructose content to induce hepatic steatosis. Lean and steatotic liver allografts, with or without multidrug donor preconditioning (MDDP), were subsequently transplanted into lean recipients to investigate their effects on IRI. Our findings indicate that MDDP treatment effectively improved the transaminase levels of steatotic rat allograft on POD7 and recipient survival following rat OLT.

## Materials and Methods

### The Ethics and Source of Animal Used in the Present Study

Male Sprague-Dawley rats weighing between 270 and 350 grams were procured from Charles River, Sulzfeld, Germany. These rats were accommodated in conventional cages under standard laboratory conditions at a temperature of 23°C ± 2°C, with a 12-hour light-dark cycle, ensuring their welfare and care aligned with the principles outlined in the “Guide for the Care and Use of Laboratory Animals” by the National Academy of Sciences, as published by the National Institutes of Health. Ethical approval for all experiments was obtained following the regulations and guidelines of the Georg-August-University of Göttingen (UMG).

### Western-Style Food and the Development of Rat Fatty Liver Model

Rats were randomly divided into two dietary groups: a normal chow food group and a high-fat and fructose (Lieber-DeCarli, LDC) diet group. Approximately 90% of the total energy intake (J) was derived from the LDC diet, with the remaining 10% J coming from fructose, replacing a portion of the maltodextrin included in the LDC diet. The LDC diet, provided in powder form, was obtained from Ssniff Spezialdiaeten GmbH, Soest 59494, Germany. The proportions of energy from protein, fat, and carbohydrates were consistent with our previous reports [22, 23]. Animals were provided with pre-weighed food in bottles *ad libitum* for a period of 30 days.

**FIGURE 1:**
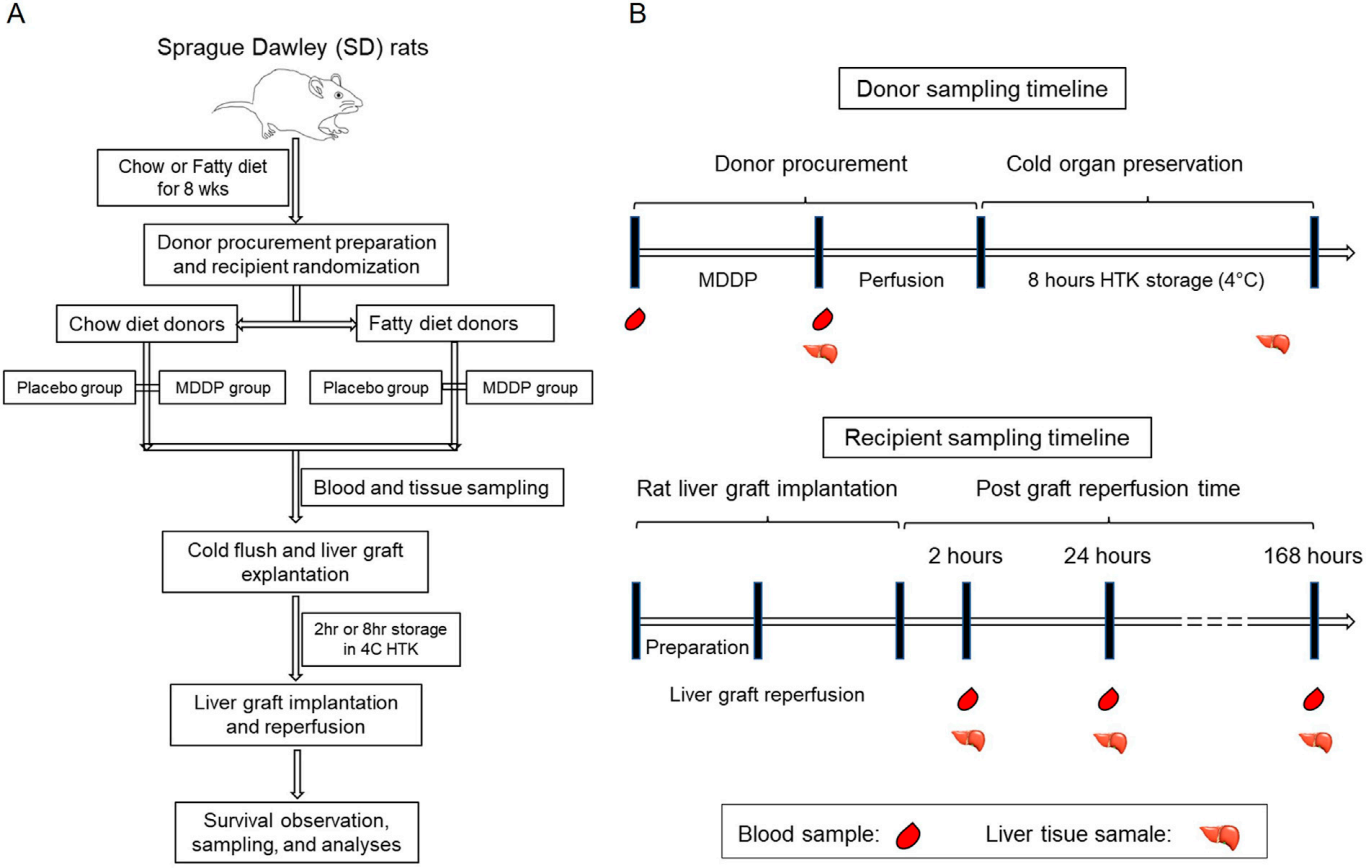
Overall experimental design of the present study. **(A)** The flow chart of rat fatty liver model fed with chow or high fructose + high fat (HF + HFr) diets, multidrug donor preconditioning (MDDP) treatment, and rat orthotopic liver transplantation (OLT). **(B)** The rat blood and liver samples collection plan.

Various combinations of cold ischemia time (CIT) and graft perfusion site were chosen to induce specific degrees of graft injury while maintaining an acceptable survival rate. This was done with the aim of developing an optimized model for steatotic rat liver transplantation for further research. The rats were divided into four groups: lean liver donors, lean donors with multidrug donor preconditioning (MDDP) treatment, fatty liver donors, and fatty liver donors with MDDP treatment (Figure 1A).

### The Perioperative Donor Multidrug Donor Preconditioning (MDDP), Procurement, and Cold Storage

The animal underwent anesthesia with a flow rate of 1.5 L/min oxygen and 4% Sevoflurane for induction, followed by maintenance with 2% Sevoflurane. The conception of MDDP was derived from our former colleagues (Prof. Dr. Kollmar, et al) in two *ex vivo* studies. Specifically, simvastatin was utilized to lower hepatic cholesterol levels by inhibiting HMG-CoA reductase and increasing eNOS and heme oxygenase 1 expression [24]; curcumin [25], N-acetylcysteine [26], erythropoietin [27], and melatonin [28] acted against oxidation; erythropoietin [27], pentoxifylline [29], and glycine inhibited cytokine [30] release; curcumin [31], erythropoietin [27], and pentoxifylline [32] inhibited apoptosis; and methylprednisolone inhibited inflammation at various stages [33, 34]. Details regarding the routes and timing of MDDP treatment are outlined in Table 1.

**TABLE 1:** The detail of multidrug donor preconditioning (MDDP) in the present study. The MDDP dosage, administration routes, and timing, together with the potential mechanisms of action.

Medication	Dosage	Administration route	Administration time	Mechanism of action
Curcumin	50 mg/kg	Intragastric (i.g.)	30 min prior liver HTK cold flush (4°C)	Anti-oxidation and apoptosis; activates HSP
Simvastatin	5 mg/kg			HMG-CoA reductase inhibitor, lowering hepatic cholesterol
N-acetylcysteine	150 mg/kg	Intraperitoneal (i.p.)		Anti-oxidation
Erythropoietin	3000 IU/kg			Inhibit oxidation, apoptosis, and TNFα production, stimulating eNOS expression
Pentoxifylline	50 mg/kg			Inhibits TNFα, Leukocytes recruitment, and apoptosis
Melatonin	10 mg/kg			Anti-oxidation
Glycine	100 mg/kg	Intravenous (i.v.)	10 min prior to liver HTK cold flush (4°C)	Attenuates Kupffer cell activation
Methylprednisolone	5 mg/kg			Anti-inflammation

The animal’s abdomen was shaved and secured to the operating table with tape. A transverse and midline incision was made in the abdomen to fully expose the liver. A bile duct stent was inserted, and branches of the portal vein (PV) and right adrenal vein/renal artery/renal vein were ligated. The inferior caudate lobe was ligated using 6-0 silk thread, resected, and then fixed in 10% neutral formalin for histological analysis. Subsequently, the abdominal artery (AA) was dissected, and 300 units of heparin were injected through the dorsal vein of the penis. Following this, 20 mL of HTK solution (Custodiol) was infused through the AA under a pressure of 10 cm H_2_O until the entire liver turned uniformly yellow. The excised livers were stored in 4°C HTK solution for 2 or 8 h prior to implantation. During cold storage, cuffs were made for the PV and infrahepatic inferior vena cava (IHIVC).

### Rat Orthotopic Liver Implantation, Samples Collection, *In Vivo* Microscopic Study, and Recipient Survival Follow-Up

The procedure for preparing and exposing the recipient’s liver followed similar steps as described for the donor operation. Once the liver was completely freed, 3 mL of normal saline and 10 units of heparin were injected intravenously. Concurrently, livers from the donors were flushed with 10 mL of normal saline. The IHIVC, PV, and suprahepatic inferior vena cava (SHIVC) were occluded. The SHIVC was anastomosed by running a 7-0 suture. Cuffs for the PV and IHIVC were inserted into the recipient’s vessels and secured with circumferential 6-0 silk sutures. The bile duct cannula of the graft was also connected. The abdominal cavity was flushed with 42°C normal saline, and a running suture was used to close it. Post-surgery, hydration of recipients, volume supplementation, and warm-up procedures were considered critical, as described in previous studies [35]. Additionally, metabolic acidosis was observed after the operation due to the clamping of the portal vein and inferior hepatic vena cava. To address this, recipient rats in this study were administered a dose of intravenous 0.5 mL bicarbonate along with 1.5 mL normal saline to improve their behavior. Subcutaneous injection of analgesia (Buprenorphine, 0.05 mg/kg) continued until the 3rd day post-operation, and a solution of 1 mL metamizole was added to the drinking water (100 mL) until the 7th day post-operation. Samples were collected as depicted in Figure 1B. The *in vivo* microscopic study was conducted as previously described [36] on post-operation day (POD) 1, and recipient survival status was checked daily until POD 7 after OLT. All recipient rats were fed a chow diet following transplantation.

### Blood Chemistry Assays for the Liver Function Panel

The measurement of serum alanine aminotransferase (ALT), aspartate aminotransferase (AST), and lactate dehydrogenase (LDH) activity, serving as biomarkers for liver injury, was conducted in the core laboratory of our institute. Furthermore, markers for biliary injury and obstruction, such as γ-glutamyl transpeptidase (GGT) and alkaline phosphatase (ALP) activity (non-specific), along with liver function tests (albumin and bilirubin levels, and prothrombin time), were analyzed. Additionally, serum samples were assessed for total lipid profile (triglycerides, HDL-cholesterol, LDL-cholesterol) and ferritin levels during the first 7 days post-transplantation using automated systems in the Department of Clinical Chemistry at the University Medical Center Göttingen, Germany.

### Histopathological Studies of Hepatic Steatosis and Reperfusion Injuries and Image Interpretation

The collected liver tissue was Formalin-fixed and paraffin-embedded (FFPE). Subsequently, the FFPE tissue specimens were sectioned into serial slices measuring 5 μm in thickness using a microtome. These sections were then deparaffinized in xylene, followed by rehydration through a graded series of ethanol, and subsequently stained with H&E. After mounting using xylene-based media, the slides were examined under a light microscope (Olympus BX43) equipped with an internal digital camera (Olympus DP21). Hematoxylin stained the nuclei blue-purple, while the cytoplasms were nonspecifically counterstained pink-red with eosin. The H&E-stained liver sections were evaluated for steatosis, hepatic vacuolization, apoptosis, and necrosis in a blinded manner.

### Data Presentation and Statistical Analysis

All the numeric data were presented in the format of mean ± standard deviation (SD). The statistical analysis was performed using the Student’s t-test with the setting of 2-tailed distribution and 2-sample equal variance. The difference was considered significant when the *p*-values were less than 0.05.

## Results

### Macroscopic and Microscopic Features of Diet-Induced Rat Fatty Liver Donors

**FIGURE 2:**
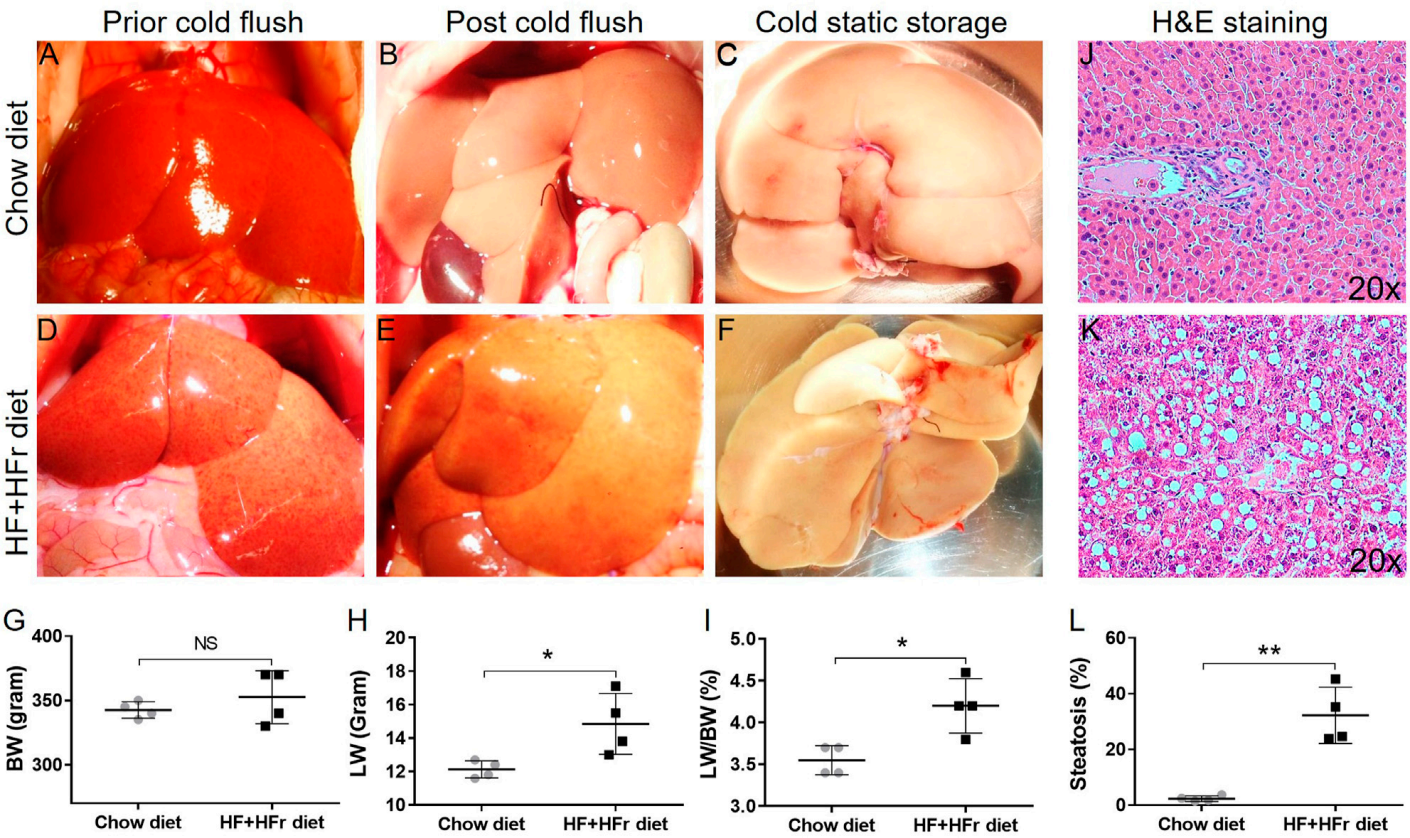
Macroscopic and microscopic features of rat liver allografts fed with chow and HF + HFr diets. The gross appearance of lean **(A–C)** and steatotic **(D–F)** rat liver allografts prior, post-HTK perfusion, and in cold storage. The body weight [BW, **(G)**], liver weight [LW, **(H)**], and LW/BW ratio **(I)** of lean and steatotic groups. The H&E staining images (×20) of lean **(J)** and fatty **(K)** rat liver allografts and quantitative analyses of steatosis [**(L)**, n = 4 per group; *p < 0.05 or **p < 0.01, t-Test].

During the explantation procedure, rat livers from the chow diet group exhibited a uniform red coloration with sharp hepatic edges (Figures 2A–C), while those from the HF + HFr diet group for 4 weeks appeared pale with interspersed red spots (Figures 2D–F). Despite no significant difference in body weight (BW, Figure 2G), both liver weight (LW, Figure 2H, *p = 0.029) and LW/BW ratio (Figure 2I, *p* = 0.012) were markedly increased in the HF + HFr diet group compared to the chow diet group. Moreover, histological examination using HE staining (Figures 2J, K) revealed a significantly higher percentage of steatosis in the HF + HFr diet group compared to the chow diet group (Figure 2L, **p = 0.001). Notably, no evidence of inflammatory infiltration or fibrosis was observed in either the livers from the HF + HFr or chow diet groups. These results suggest that the HF + HFr diet successfully induced steatotic liver in rat model.

### The Blood Chemistry of Lean and Steatotic Rat Liver Donors

**FIGURE 3:**
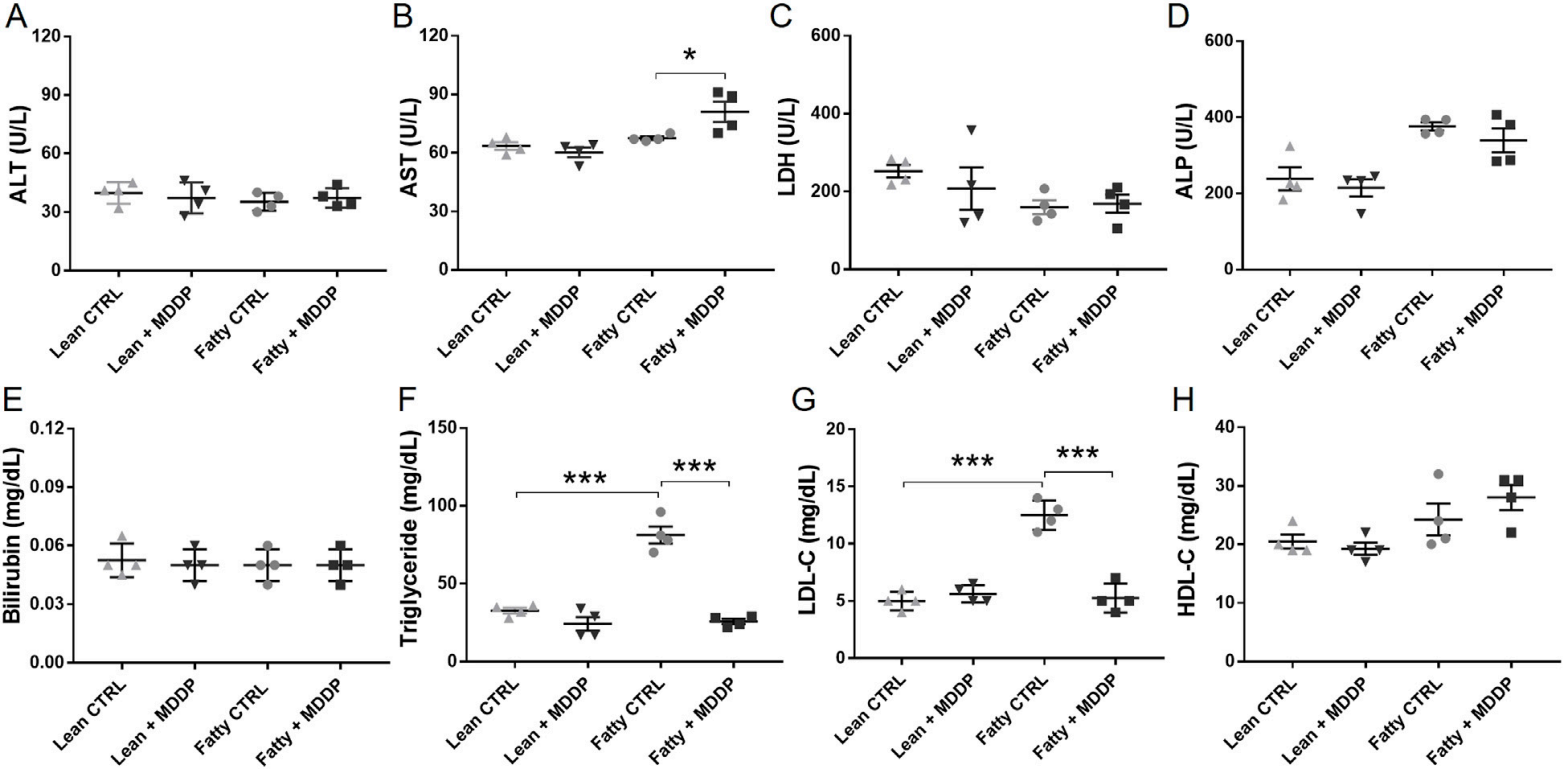
The clinical chemistry parameters of rat liver donor blood samples. The liver function panel includes ALT **(A)**, AST **(B)**, LDH **(C)**, ALP **(D)**, Bilirubin **(E)**, Triglyceride **(F)**, LDL **(G)**, and HDL **(H)** of lean and steatotic rats fed with chow or HF + HFr diets shortly before liver explantation (n = 4 per group, *p < 0.05 or ***p < 0.001, t-Test).

To assess the impact of MDDP treatment on liver function, we analyzed blood samples taken just before the cold HTH flush for various chemistry parameters, including ALT, AST, LDH, ALP, bilirubin, triglycerides, LDL, and HDL (Figure 3). While the ALT levels remained unchanged (Figures 3A, B), AST levels showed a marginally significant increase in the fatty + MDDP group compared to the fatty control group, indicating a potential hemolytic process resulting from MDDP treatment (*p* = 0.045). Additionally, triglyceride (TG) and LDL levels were notably elevated in the fatty control group compared to the lean control group (Figures 3F, G), a trend significantly mitigated by MDDP treatment (***p < 0.001, respectively). The rapid decrease in donor blood TG and LDL levels might stem from synergistic drug interactions. For instance, curcumin could enhance the effectiveness of statins in lowering cholesterol [37]. Additionally, pentoxifylline has demonstrated synergistic effects with simvastatin in cancer therapy [38]. Our findings also indicate that there were no significant differences in ALT, LDH, ALP, bilirubin, or HDL levels between the MDDP treatment group and the control group, regardless of whether the livers were lean or fatty.

### The Effects of MDDP on Rat Liver Allograft Reperfusion and Histological Findings

**FIGURE 4:**
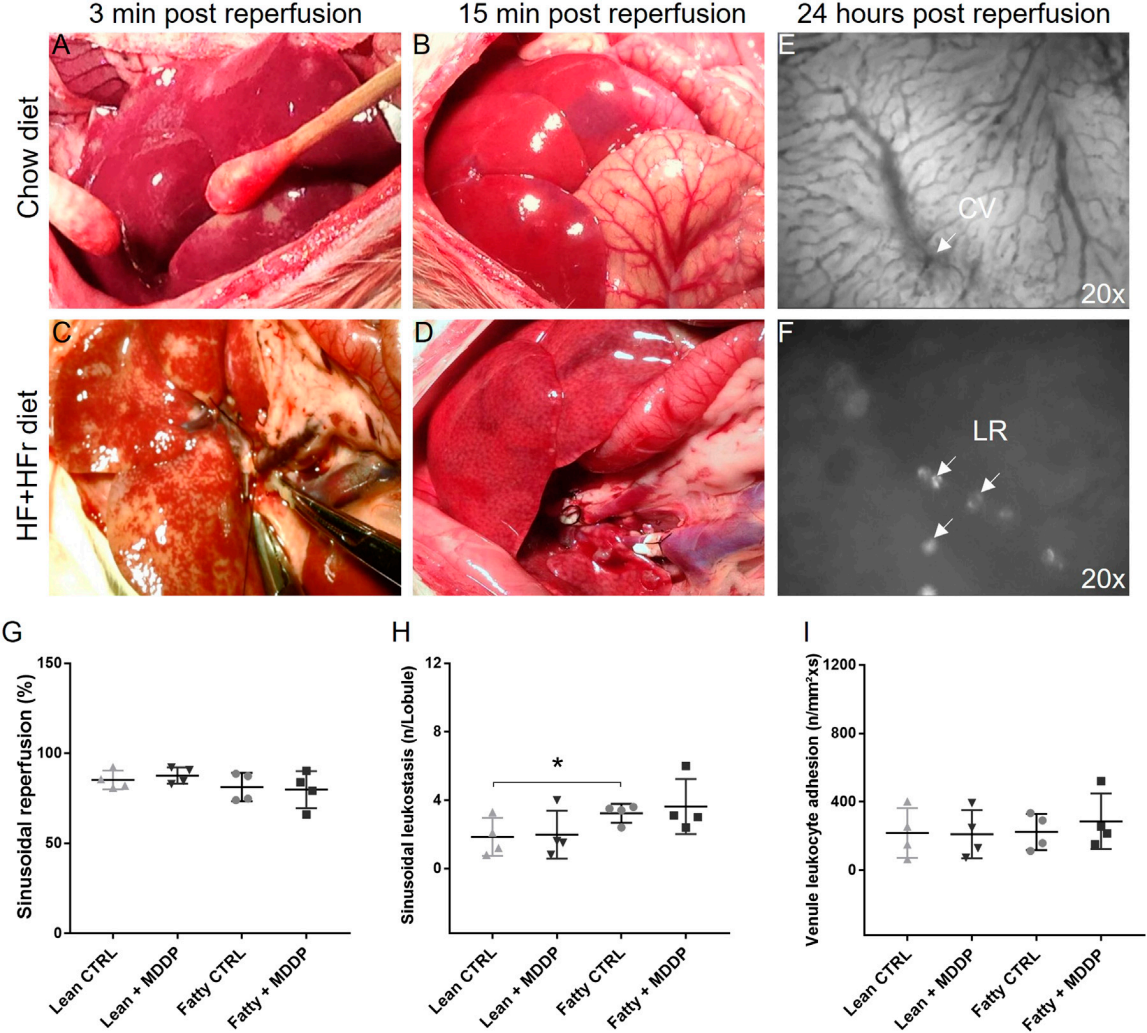
The reperfusion of rat liver allografts and *in vivo* microcirculation study. The lean **(A, B)** and steatotic **(C, D)** allografts at 3 min and 15 min after reperfusion. **(E, F)** *In vivo* microcirculation studies of the rat liver allograft 24 h after transplantation showed the hepatic sinus, central vein (CV, 20x), and Hepatic leukocyte rolling (LR, 20x). **(G–I)** The quantitative analyses of sinusoidal reperfusion **(G)**, sinusoidal leukostasis **(H)**, and venule leukocyte adhesion [**(I)**, n = 4 per group, *p < 0.05, t-Test].

We conducted a comparison of reperfusion dynamics between lean and steatotic rat liver allografts without MDDP treatment, revealing a noticeable delay in reperfusion for the steatotic livers compared to the lean ones (Figures 4A–D). Specifically, patchy areas were observed on all lean and steatotic rat livers shortly after portal reperfusion, potentially caused by small air embolisms, vasospasms, or mechanical injuries. This could be a systematic error that would not affect the statistical comparisons between the different groups. However, at the 3rd-minute post-reperfusion initiation, the steatotic liver allograft (Figure 4C) exhibited more areas of non-reperfusion compared to the lean graft (Figure 4A). By the 15th minute post-reperfusion, the steatotic rat liver (Figure 4D) displayed more dark areas compared to the lean liver (Figure 4B), indicating poorer reperfusion and potentially more severe reperfusion injury in the steatotic liver allografts than in the lean liver allografts.

**FIGURE 5:**
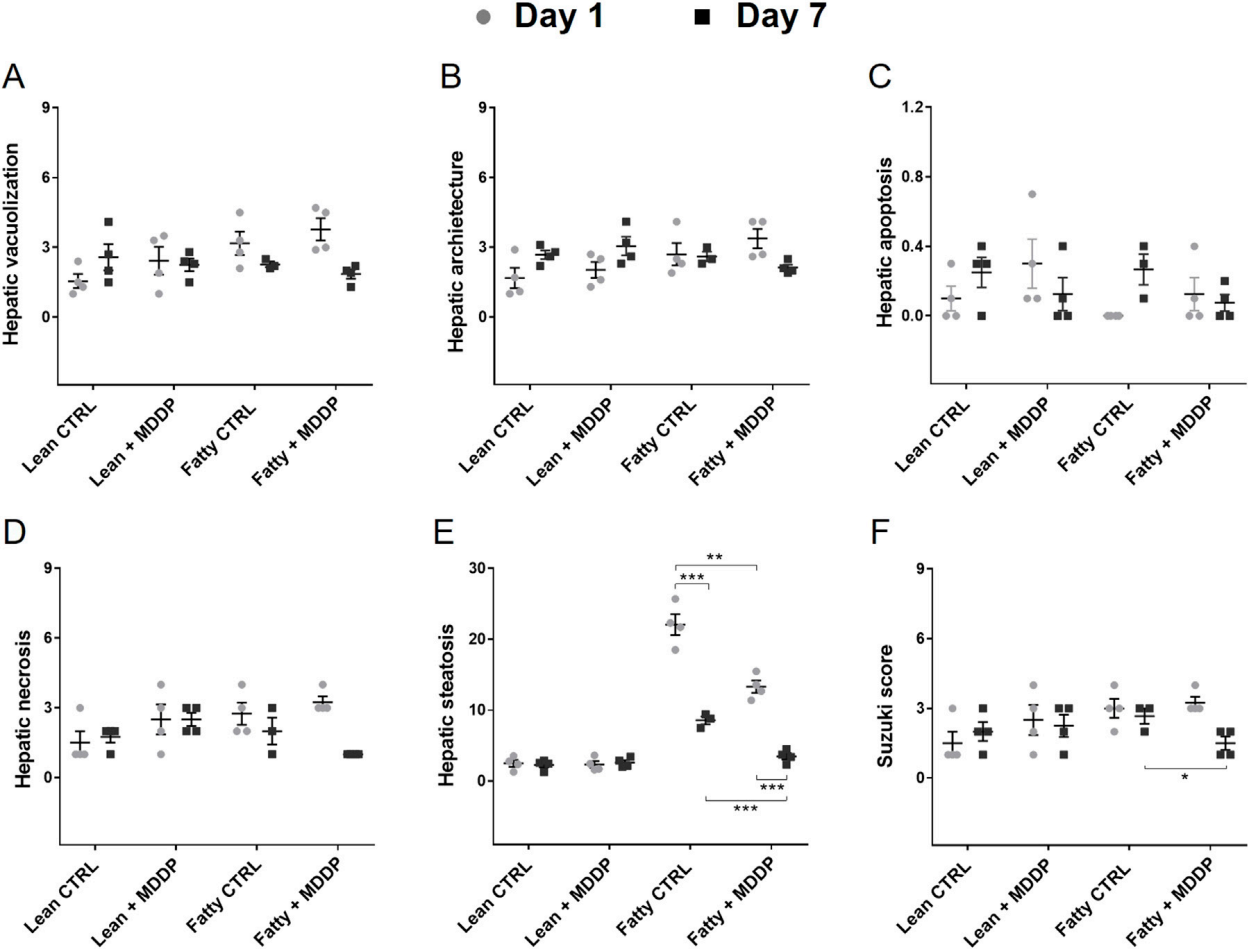
The histological studies of rat liver allografts after transplantation. Histology analyses on POD 1 and 7 after transplantation, including Hepatocyte vacuolization **(A)**, Hepatic architectures **(B)**, Hepatic apoptosis **(C)**, Hepatic necrosis **(D)**, Hepatic fat contents **(E)**, and Suzuki scores [**(F)**, n = 3 or 4 per group, *p < 0.05, **p < 0.01, or ***p < 0.001, respectively, t-Test].

Additionally, *in vivo* microscopy was employed to investigate hepatic micro-reperfusion (Figure 4E) and leukocyte status (Figure 4F) at 24 h post-rat liver OLT. Remarkably, sinusoidal leukostasis was significantly higher in the steatotic liver allografts compared to the lean liver allografts (*p* = 0.016, Figure 4H). However, MDDP treatment did not induce significant changes in sinusoidal reperfusion (Figure 4G), sinusoidal leukostasis (Figure 4H), or venule leukocyte adhesion (Figure 4I), irrespective of whether lean or steatotic rat liver allografts were transplanted, at 24 h post-transplantation. Further examination of H&E images on POD 1 and 7 revealed that MDDP treatment did not alter hepatic vacuolization, architecture, apoptosis, or necrosis in either lean or steatotic rat liver transplantation (Figures 5A–D). We further found that the hepatic fat contents in both the control and MDDP groups were significantly decreased on POD 7 than on POD 1 (Figure 5E, both ***p < 0.001, respectively). Moreover, the hepatic fat contents in the MDDP-treated rat livers appeared to be lower than that in the control group at both POD 1 and POD 7 (Figure 5E, **p = 0.002 and ***p < 0.001, respectively). In addition, the Suzuki scores were significantly lower in the MDDP group than in the control group on POD 7 (Figure 5F, *p = 0.046).

### The Recipient Rats’ Blood Chemistry and Recipient Survival

**FIGURE 6:**
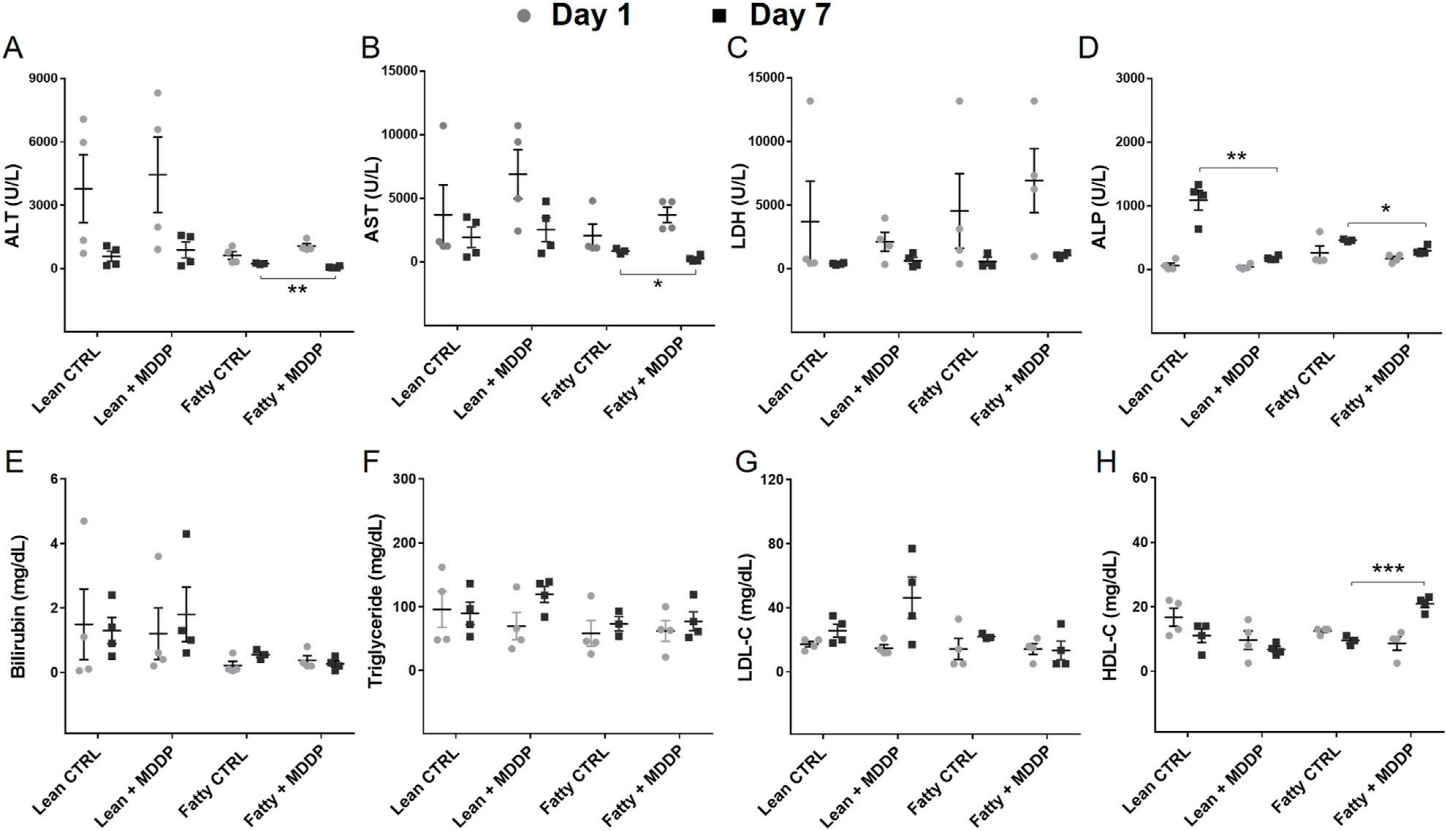
The clinical chemistry parameters of rat recipient blood samples. The liver function panel includes ALT **(A)**, AST **(B)**, LDH **(C)**, ALP **(D)**, Bilirubin **(E)**, Triglyceride **(F)**, LDL **(G)**, and HDL **(H)** of lean and steatotic rats fed with chow or HF + HFr diets on POD 1 and 7 after OLT without or with MDDP (n = 3 or 4 per group, *p < 0.05, **p < 0.01 or ***p < 0.001, t-Test).

To evaluate the impact of MDDP treatment on liver allograft function, we analyzed blood samples collected on POD 1 and 7 for various chemistry parameters. Our findings revealed a significant reduction in ALT (Figure 6A, **p = 0.003) and AST (Figure 6B, *p = 0.021) levels in the steatotic rat liver allograft group following MDDP treatment on POD 7. However, these reductions were not observed in the lean rat liver allograft group. Furthermore, MDDP treatment led to a significant decrease in ALP levels in both the steatotic rat liver allograft (*p = 0.011) and lean rat liver allograft (**p = 0.001) groups on POD 7 (Figure 6D). MDDP treatment significantly increased HDL levels in the steatotic rat liver allograft group on POD 7 (Figure 6H, ***p < 0.001). In contrast, no such effect was observed in the lean rat liver allograft group. Furthermore, the MDDP did not significantly affect the recipient’s blood levels of LDH (Figure 6C), bilirubin (Figure 6E), triglycerides (Figure 6F), or LDL (Figure 6G) on POD 1 and POD 7.

**FIGURE 7:**
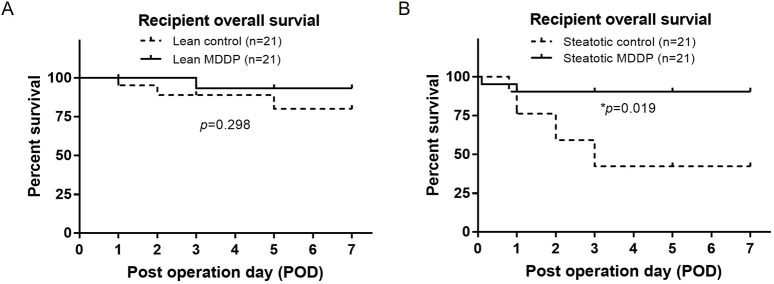
The rat recipient’s survival time after orthotopic liver transplantation (OLT). Rat recipient survival after OLT of lean **(A)** and steatotic **(B)** liver allografts with 8 h of cold ischemia and without or with MDDP treatment (n = 21 per group, *p < 0.05, Log-rank test).

In our studies, recipients of lean rat liver donors with a 2-hour CIT had an AST level of 318.4 ± 81.4 U/L on POD1 and achieved nearly 100% recipient survival over 3 months (n = 10). As depicted in Figure 7, no significant difference in recipient survival was observed between the control group (18/21, 85.7%) and the MDDP group (20/21, 95.2%) using lean donors with 8 h CIT (Figure 7A, *p* = 0.298, Log-rank test). However, the survival rates of steatotic rat liver allografts with 8 h of CIT and MDDP treatment (19/21, 90.5%) were significantly higher than those in the steatotic control group (12/21, 57.1%, Figure 7B, *p = 0.019, Log-rank test). These findings suggest that MDDP treatment improves steatotic rat liver allograft function and recipient survival following OLT.

## Discussion

The acceptance of steatotic liver donors for transplantation in patients with end-stage liver disease has risen, yet these liver allografts exhibit heightened vulnerability to ischemia-reperfusion injury (IRI) post-OLT. In addressing this challenge, we employed a multidrug donor preconditioning approach to mitigate IRI in steatotic rat liver allografts following OLT in a rat model. Our study revealed that MDDP treatment significantly improved the function of steatotic rat liver allograft and recipient survival post-transplantation.

We first successfully induced a rat steatotic liver model by administering a Western-style diet abundant in fat and fructose as previously described [22, 23]. This induction was characterized by elevated blood triglyceride and cholesterol levels, as well as the accumulation of both microvesicular and macrovesicular fatty droplets within the hepatocytes. Subsequently, the implanted steatotic rat liver allografts exhibited delayed and uneven reperfusion, which was associated with compromised graft function and diminished survival post-OLT, which are consistent with previous studies [39, 40]. This model provided an excellent platform for investigating strategies aimed at mitigating the risks associated with steatotic liver donor transplantation. Notably, the MDDP treatment significantly reduced donor blood triglyceride and cholesterol levels without impacting liver-specific enzymes, indicating a favorable therapeutic outcome without notable hepatotoxic effects. Interestingly, we also observed a mild increase in blood AST levels in the steatotic donors with MDDP treatment compared to the steatotic control group, which may be due to an increased hemolytic process. It has been revealed that a strong positive correlation between blood cholesterol levels and RBC rigidity could affect the cell membrane fluidity, thus affecting the deformability of erythrocytes [41]. The RBC deformability can be further increased by an increase of RBC membrane cholesterol content in response to the lipid-lowering drug simvastatin, which can result in an increased risk of hemolysis [42]. However, the evaluation of donor blood AST levels associated with MDDP treatment was relatively mild.

The IRI of liver allografts involves a multifaceted process characterized by various pathways, including the exacerbation by steatosis of reactive oxygen species (ROS), the release of proinflammatory cytokines by activated Kupffer cells, and occurrences of leukocyte adhesion, vasoconstriction, apoptosis, and necrosis [43–46]. Given the complexity of IRI and the involvement of numerous molecular pathways, we employed a pharmacological combination of multidrug donor conditioning aimed at multiple pathways in allograft IRI following OLT. Furthermore, we employed a prolonged cold ischemia time (CIT) to induce significant liver damage, allowing us to assess the therapeutic effects of MDDP treatment. As expected, on POD1, there was a marked elevation of transaminase levels in the lean and steatotic controls with 8 h CIT compared to the lean control with 2 h CIT. Elevated AST levels exceeding 7500 U/L on POD1 have been associated with reduced recipient survival following OLT [47]. Although not statistically significant, the overall transaminase levels appeared paradoxically lower in the steatotic group (Control + MDDP) with 8 h CIT compared to the lean group (Control + MDDP) under the same conditions. This suggests that lean and steatotic livers may respond differently to prolonged CIT. The exact mechanism behind the relatively lower transaminase levels in the steatotic control group on POD 1 remains unclear. It is speculated, however, that steatotic hepatocytes may have reduced transaminase reserves due to impaired synthetic function [48], similar to the drop in transaminase levels observed in patients with liver failure [49]. Interestingly, we observed a significant reduction in blood transaminase levels and hepatic Suzuki scores on POD 7, along with improved survival in recipients of steatotic rat liver grafts treated with MDDP compared to those who did not receive MDDP treatment. However, these effects were not significant in lean rat liver allograft transplantation, suggesting the benefits of this MDDP treatment may be limited in steatotic liver transplantation.

Another noteworthy finding was the significant decrease in steatosis of rat liver grafts following OLT on POD 1, which was further reduced with MDDP treatment. This finding aligns with previous studies, which show that 4–8 h of normothermic machine perfusion (NMP) with a defatting solution can reduce hepatic steatosis by up to 40% in discarded human livers [50, 51] and rat fatty liver models [20, 21]. In addition, no significant histological changes, including hepatic inflammation, apoptosis, and necrosis, were observed at 24 h post-transplantation, potentially attributable to the inappropriate timing of tissue examination as the hepatic necrosis may occur up to 48 h after reperfusion [52–54].

A notable limitation of this study is the difficulty in identifying the precise mechanism by which MDDP treatment improves rat liver allograft function and survival post-transplantation, due to the complex nature of ischemia-reperfusion injury and the multifaceted mechanisms of action of the drug combination. Moreover, our study is also limited by a lack of translational capacity, as it would be unrealistic to deliver intra-gastric and intra-peritoneal medications during human organ procurement. The potential effects of the MDDP on other organs remain unknown. Further studies with *ex vivo* normothermic liver machine perfusion and transplantation are required to validate the efficacy and possible toxicity of MDDP treatments.

In summary, we established a diet-induced steatotic rat liver transplantation model with satisfactory liver damage and survival rate after OLT, enabling further exploration in pharmacological studies. Our findings demonstrate that MDDP treatment effectively improved the steatotic rat liver allograft function and recipient survival following transplantation.

## Data Availability

The raw data supporting the conclusions of this article will be made available by the authors, without undue reservation.

## Abbreviations

OLT, Orthotopic liver transplantation; MDDP, Multidrug donor preconditioning; IRI, Ischemia-reperfusion injury.
